# Early Coagulation Disorder Is Associated With an Increased Risk of Atrial Fibrillation in Septic Patients

**DOI:** 10.3389/fcvm.2021.724942

**Published:** 2021-09-30

**Authors:** Yunxiang Long, Yingmu Tong, Runchen Miao, Rong Fan, Xiangqi Cao, Jie Wang, Jingjing Sun, John D. Day, Chang Liu, Guoliang Li

**Affiliations:** ^1^Department of Hepatobiliary Surgery, The First Affiliated Hospital of Xi'an Jiaotong University, Xi'an, China; ^2^Department of SICU, The First Affiliated Hospital of Xi'an Jiaotong University, Xi'an, China; ^3^Department of Cardiovascular Medicine, The First Affiliated Hospital of Xi'an Jiaotong University, Xi'an, China; ^4^Stroke Centre and Department of Neurology, The First Affiliated Hospital of Xi'an Jiaotong University, Xi'an, China; ^5^Department of Hematology, The First Affiliated Hospital of Xi'an Jiaotong University, Xi'an, China; ^6^Department of Critical Care Medicine, The First Affiliated Hospital of Xi'an Jiaotong University, Xi'an, China; ^7^Department of Cardiology, St. Mark's Hospital, Salt Lake City, UT, United States

**Keywords:** neurocardiovascular, atrial fibrillation, coagulation disorder, ischemic stroke, sepsis

## Abstract

**Background:** Atrial fibrillation (AF) and coagulation disorder, two common complications of sepsis, are associated with the mortality. However, the relationship between early coagulation disorder and AF in sepsis remains elusive. This study aimed to evaluate the interaction between AF and early coagulation disorder on mortality.

**Methods:** In this retrospective study, all data were extracted from the Medical Information Mart for Intensive Care III (MIMIC-III) database. Septic patients with coagulation tests during the first 24 h after admission to intensive care units (ICUs) meeting study criteria were included in the analysis. Early coagulation disorder is defined by abnormalities in platelet count (PLT), international normalized ratio (INR) and activated partial thromboplastin time (APTT) within the first 24 h after admission, whose score was defined with reference to sepsis-induced coagulopathy (SIC) and coagulopathy. Patients meeting study criteria were divided into AF and non-AF groups.

**Results:** In total, 7,528 septic patients were enrolled, including 1,243 (16.51%) with AF and 5,112 (67.91%) with early coagulation disorder. Compared with patients in the non-AF group, patients in the AF group had higher levels of INR and APTT (*P* < 0.001). Multivariable logistic regression analyses showed that stroke, early coagulation disorder, age, gender, congestive heart failure (CHF), chronic pulmonary disease, renal failure, and chronic liver disease were independent risk factors for AF. In addition, AF was related to in-hospital mortality and 90-day mortality. In the subgroup analysis stratified by the scores of early coagulation disorder, AF was associated with an increased risk of 90-day mortality when the scores of early coagulation disorder were 1 or 2 and 3 or 4.

**Conclusion:** In sepsis, coagulation disorder within the first 24 h after admission to the ICUs is an independent risk factor for AF. The effect of AF on 90-day mortality varies with the severity of early coagulation disorder.

## Introduction

In sepsis, AF is the most common arrhythmia, with an incidence of 20–30% (1). High levels of circulating stress hormones and inflammatory cytokines, autonomic nervous system dysfunction, blood volume change, and cardiovascular injury promote atrial structure and electrical remodeling to act as substrates of AF (2). Therefore, patients accompanied with AF have more thrombosis, longer hospital stay, as well as a higher risk of death, especially in severe cases (3–6).

Coagulation dysfunction, including SIC and DIC, are common complications in sepsis (7). After being triggered by an acute systemic inflammatory response of sepsis, coagulation initiates and induces extensive crosstalk with inflammation and immunity to promote SIC and DIC (8, 9). This process results in massive consumption of coagulation substances, then pathological coagulation occurs to further damage the vascular endothelial barrier and contribute to micro thrombosis and micro bleeding (10). These pathophysiologic mechanisms contributing to multiple system organ ischemia and ischemia-reperfusion injury (11) are, in part, related to a high risk of cardiovascular events. Therefore, SIC is proposed to identify the earlier stage of coagulation abnormalities in sepsis.

Based on preclinical findings, hyper coagulant-transgenic animals were more likely to have AF than wild-type animals, suggesting that hypercoagulation may cause or promote AF (12). However, it remains elusive whether abnormal coagulation is a risk factor for AF in the real world. Therefore, the present work aimed to evaluate the effects between early coagulation disorder and AF on the mortality in sepsis.

## Materials and Methods

### Study Design

The retrospective study was performed with data from the Medical Information Mart for Intensive Care-III (MIMIC-III) database, consisting of data on 53,423 different hospital admissions for adult patients presenting to the ICUs between 2001 and 2012. MIMIC-III is a large, freely available database comprising de-identified health-related data associated with patients admitted to the critical care units of Beth Israel Deaconess Medical Center. As a result, the informed consent and approval of the Institutional Review Board were waived. Data were not eligible for access until approved by MIT's (Massachusetts Institute of Technology) institutional review board. All data were extracted by an author (Record ID: 28572693) who completed the CITI “Data or Specimens Only Research” course and passed the exam.

### Patients

All septic adult patients with coagulation function tests within 24 h after admission to the ICUs were evaluated for enrollment in this study. The diagnostic criteria for sepsis were consistent with sepsis 3.0, which is defined after meeting the following conditions: patients with documented or suspected infection; an acute increase of Sequential Organ Failure Assessment (SOFA) score of ≥2. Suspected infection was identified as prescriptions of antibiotics and sampling of bodily fluids for microbiological culture (13). The exclusion criteria were age>80 years, pregnancy, congenital heart diseases, valvular heart diseases, congenital coagulation disorders (e.g., hemophilia, vitamin K deficiency), coronary artery stenosis, implanted cardiac devices, and admission to the cardiac care unit (CCU) as well as cardiac surgery intensive care unit (CSICU). If there were multiple ICU admissions in a patient, the first information was only included for analysis. Eventually, patients were divided into two groups based on the status of AF, which was recognized from the database by ICD-9 codes.

### Variables

Baseline characteristics of the patients included the following: age, gender, ethnicity, height, weight, insurance type, marital status, ICU type, mean arterial pressure (MAP), heart rate (HR), temperature (T), and respiratory rate (RR), complications including hypertension, diabetes, CHF, peripheral vascular disease, renal failure, chronic liver disease, chronic pulmonary disease, stroke, DIC, critical illness scores including Elixhauser comorbidity index (ECI), SOFA score, Acute Physiological Score III (APS III), Systemic Inflammatory Response Syndrome (SIRS) score, Simplified Acute Physiology Score II (SAPS II), Overall Anxiety Severity and Impairment Scale (OASIS) score, Glasgow Coma Score (GCS), PLT, INR, APTT, and AF. ECI, a comprehensive comorbidity scoring system with 30 comorbidities, is appropriate for studies of databases. INR reflects the standardized prothrombin time, which is a ratio of prothrombin time between patients and normality after being adjusted with the international sensitivity index (ISI). If there were several reports on a variable within the first 24 h, the worst value was chosen for analysis.

### Outcomes

The primary outcome was 90-day mortality. Secondary outcomes were the morbidity of AF, in-hospital, ICU, and 28-day mortality.

### Statistical Analysis

Variables are divided into continuous and categorical variables. Continuous variables were described as the medians with interquartile range (IQR). The Kolmogorov-Smirnov test was used to evaluate the normality of variables. Student's *t*-test and Wilcoxon Mann–Whitney test were applied to compare continuous variables between AF and non-AF groups. Categorical variables were shown as frequencies with percentages and were compared with the chi-square test or Fisher's exact test.

Additive PLT, INR, and APTT scores were used to define early coagulation disorder with reference to SIC and coagulopathy (**Table 2**) (12, 14). Propensity score matching (PSM) minimized the imbalance of AF and non-AF groups with age, gender, ICU type, ethnicity, and marital status. Univariate and multivariate logistic regression analyses were used to evaluate the association of risk factors and outcomes. Baseline variables that were considered clinically relevant or that showed a univariate relationship with the outcome were included in a multivariate logistic regression model as covariates. The interaction between early coagulation disorder and AF on mortality was assessed by multivariate logistic regression analyses.

Statistical analyses were performed using SPSS 26.0. *P* < 0.05 was statistically significant.

## Results

### Demographic and Baseline Characteristics

**Figure 1:**
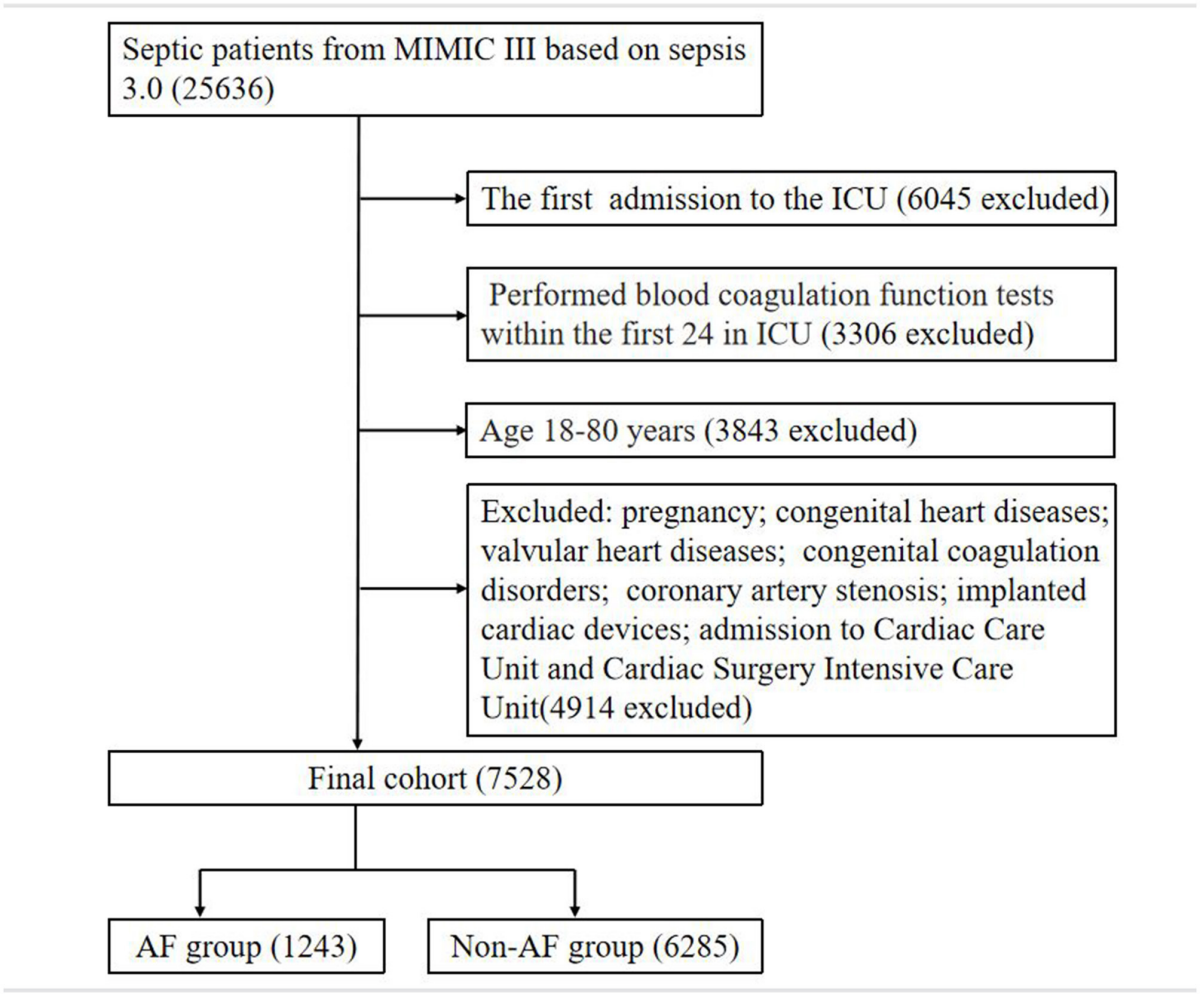
The flowchart of study patients.

Overall, 7,528 septic patients were enrolled in this study, of whom 1,243 (16.51%) had AF, and 5,112 (67.91%) had coagulation disorder (Figure 1). The median age was 61, with a range from 49 to 70 years old. Female patients comprised 43.65% of the total. The demographic data between the AF group and non-AF group are described (Table 1). The patients accompanied with AF were older than that of patients without AF (70, 63–76 vs. 58, 48–69; *P* < 0.001). Furthermore, stroke (0.76 vs. 3.30%; *P* < 0.001), CHF (16.23 vs. 41.43%; *P* < 0.001), hypertension (43.90 vs. 56.48%; *P* < 0.001), diabetes (24.47 vs. 35.32%; *P* < 0.001), ECI (11, 5–17 vs. 17.12–22; *P* < 0.001), APS III (49, 37–63 vs. 53, 42–67; *P* < 0.001), INR (1.3, 1.2–1.7 vs. 1.5, 1.2–2.2; *P* < 0.001), and APTT (32.7, 27.7–43.9, vs. 34.6, 29–46.1; *P* < 0.001) were significantly different in patients between the AF and non-AF groups. Among septic patients, the AF group had higher in-hospital mortality and 90-day mortality (19.71 vs. 13.79%, *P* < 0.001; 59.21 vs. 56.04%, *P* = 0.001). After 1:1 PSM, consisting of 1039 septic patients with AF and 1039 matched individuals without AF as controls, differences in INR, APTT and 90-day mortality between two groups still remained (*P* < 0.05; Supplementary Table 1).

**Table 1:** Demographic characteristics between AF and Non-AF groups within the first 24 h after admission to ICUs before PSM.

**Variables**	**Total**	**Non-AF**	**AF**	***P*-value**
	***n* = 7,528**	***n* = 6,285**	***n* = 1,243**	
Age (years)	61 (49–70)	58 (48–69)	70 (63–76)	<0.001
Female, (*n*, %)	3,286 (43.65)	2,775 (42.41)	511 (41.11)	0.048
**Ethnicity**, ***n*** **(%)**				0.001
Asian	225 (2.99)	192 (3.05)	33 (2.65)	
Black	773 (10.27)	667 (10.61)	773 (8.53)	
Hispanic	316 (4.20)	281 (4.47)	316 (2.82)	
White	5,333 (70.84)	4,391 (69.86)	5,333 (75.78)	
Other	881 (11.70)	754 (12.00)	881 (10.22)	
BMI (kg/m^2^)	27.7 (23.9–33.0)	27.6 (23.7–32.8)	28.7 (24.7–34.1)	<0.001
**Insurance type**, ***n*** **(%)**				<0.001
Government	269 (3.57)	256 (4.07)	13 (1.05)	
Medicaid	967 (12.85)	913 (14.53)	54 (4.34)	
Medicare	3,479 (46.21)	2,590 (41.20)	889 (71.52)	
Private	2,707 (35.96)	2,428 (38.63)	279 (22.44)	
Self-pay	106 (1.41)	98 (1.56)	8 (0.64)	
**Marital status**, ***n*** **(%)**				<0.001
Single	2,394 (36.92)	2,118 (38.91)	276 (26.51)	
Married	3,442 (53.08)	2,797 (51.38)	645 (61.96)	
Divorced	552 (8.51)	447 (8.21)	105 (10.09)	
Other	97 (1.50)	82 (1.51)	15 (1.44)	
**ICU type**, ***n*** **(%)**				<0.001
SICU	1,683 (22.36)	1,408 (22.40)	275 (22.12)	
TSICU	1,242 (16.50)	1,089 (17.33)	153 (12.31)	
MICU	4,603 (61.15)	3,788 (60.27)	815 (65.57)	
**Vital signs**				
HR (bpm)	90 (78–102)	91 (79–102)	89 (77–102)	0.056
RR (bpm)	19 (17–22)	19 (17–22)	20 (17–23)	<0.001
T (°C)	36.9 (36.5−37.4)	37.0 (36.5–37.5)	36.9 (36.4–37.3)	<0.001
MAP (mmHg)	77 (70–85)	77 (70–85)	75 (68–82)	<0.001
**Complications**, ***n*** **(%)**				
Hypertension	3,463 (45.98)	2,759 (43.90)	702 (56.48)	<0.001
Diabetes	1,977 (26.26)	1,538 (24.47)	439 (35.32)	<0.001
CHF	1,534 (20.39)	1,019 (16.23)	515 (41.43)	<0.001
Peripheral vascular disease	411 (5.46)	300 (4.78)	111 (8.93)	<0.001
Renal failure	1,127 (14.98)	818 (13.03)	309 (24.86)	<0.001
Chronic liver disease	1,506 (20.02)	1,340 (21.34)	166 (13.35)	<0.001
Chronic pulmonary disease	1,569 (20.86)	1,196 (19.05)	373 (30.00)	<0.001
Stroke	89 (1.18)	48 (0.76)	41 (3.30)	<0.001
DIC	179 (2.38)	151 (2.40)	28 (2.25)	0.206
**Critical illness score**				
ECI	11 (5-17)	11 (5-17)	17 (12–22)	<0.001
SOFA	5 (3-7)	5 (3-7)	5 (3-7)	<0.001
APSIII	47 (35–62)	49 (37–63)	53 (42–67)	<0.001
SAPSII	36 (28-46)	37 (29–46)	41 (34–51)	<0.001
OASIS	33 (27–39)	33 (27–39)	35 (29–42)	<0.001
GCS	15 (13–15)	15 (14–15)	15 (14–15)	0.547
**Coagulation function**				
PLT(10^9^/L)	179 (109–256)	168 (97–251)	179 (107–256)	0.102
INR	1.3 (1.1–1.7)	1.3 (1.2–1.7)	1.5 (1.2–2.2)	<0.001
APTT (s)	31.5 (27–40.5)	32.7 (27.7–43.9)	34.6 (29–46.1)	<0.001
**Outcomes**				
ICU morality, *n* (%)	785 (10.43)	626 (9.96)	159 (12.79)	0.003
In-hospital mortality, *n* (%)	1,112 (14.77)	867 (13.79)	245 (19.71)	<0.001
28-day mortality, *n* (%)	1,236 (16.41)	968 (15.40)	268 (21.56)	<0.001
90-day mortality, *n* (%)	4,528 (56.56)	3,522 (56.04)	736 (59.21)	0.001

*AF, atrial fibrillation; BMI, body mass index; SICU, surgical intensive care unit; TSICU, trauma surgical intensive care unit; MICU, medical intensive care unit; HR, heart rate; RR, respiratory rate; T, temperature; MAP, mean arterial pressure; CHF, congestive heart failure; DIC, disseminated intravascular coagulation; ECI, Elixhauser comorbidity index; SOFA, sequential organ failure assessment; APSIII, acute physiological score III; SAPSII, simplified acute physiology score II; OASIS, overall anxiety severity and impairment scale; GCS, Glasgow coma score; PLT, platelet count; INR, international normalized ratio; APTT, activated partial thromboplastin time*.

### Associations Between Early Coagulation Disorder and Atrial Fibrillation

There were more abnormal values of coagulation variables in the AF group (*P* < 0.05). Therefore, early coagulation disorder was classified by adding the PLT, INR, and APTT scores (Table 2).

**Table 2:** Comparisons of early coagulation disorder between AF group and Non-AF group before PSM.

**Coagulation**	**Total**	**Non-AF**	**AF**	***P*-value**
**PLT score**				0.002
0 (≥150 × 10^9^/L)	4,508 (59.88)	3,733 (59.40)	775 (62.35)	
1 (100–150 × 10^9^/L)	1,440 (19.13)	1,186 (18.87)	254 (20.43)	
2 (<100 × 10^9^/L)	1,580 (20.99)	1,366 (21.73)	214 (17.22)	
**INR score**				<0.001
0 ( ≤ 1.2)	4,198 (55.77)	3,664 (58.30)	534 (42.96)	
1 (1.2-1.4)	1,587 (21.08)	1,325 (21.08)	262 (21.08)	
2 (>1.4)	1,743 (23.15)	1,296 (20.62)	447 (35.96)	
**APTT score**				<0.001
0 ( ≤ 37 s)	5,153 (68.45)	4,387 (69.80)	766 (61.62)	
1 (37–39 s)	290 (3.85)	227 (3.61)	63 (5.07)	
2 (>39 s)	2,085 (27.70)	1,671 (26.59)	414 (33.30)	
**Total score**		1 (0–3)	2 (1–4)	<0.001
**Coagulation disorder type**				<0.001
Coagulation disorder score = 0	2,416 (32.09)	2,132 (33.92)	284 (22.85)	
Coagulation disorder score = 1 or 2	2,708 (35.97)	2,231 (35.50)	477 (38.37)	
Coagulation disorder score = 3 or 4	1,591 (21.13)	1,242 (19.76)	349 (28.08)	
Coagulation disorder score = 5 or 6	813 (10.80)	680 (10.82)	133 (10.70)	

*AF, atrial fibrillation; PLT, platelet count; INR, international normalized ratio; APTT, activated partial thromboplastin time*.

Multivariate logistic regression analyses (Table 3) showed that the incidence of AF was associated with the following: stroke (adjusted OR = 3.756; 95% CI: 2.269–6.215; *P* < 0.001), coagulation disorder types (early coagulation disorder type = 1; as reference; *P* < 0.001), age (adjusted OR = 1.072; 95% CI: 1.064–1.080; *P* < 0.001), gender (adjusted OR = 1.276; 95% CI: 1.096–1.487; *P* = 0.002), CHF (adjusted OR = 2.378; 95% CI: 2.022–2.797; *P* < 0.001), chronic pulmonary disease (adjusted OR = 1.350; 95% CI: 1.139–1.600; *P* = 0.001), renal failure (adjusted OR = 1.459; 95% CI: 1.203–1.769; *P* < 0.001) and chronic liver disease (adjusted OR = 0.664; 95% CI: 0.531–0.829; *P* < 0.001). After 1:1 PSM, early coagulation disorder was still an independent risk factor for AF (reference as early coagulation disorder type = 1; *P* < 0.001).

**Table 3:** Association between early coagulation disorder and AF in multivariate logistic regression before and after PSM.

**Variables**	**Before PSM**		**After PSM**	
	**OR; 95% CI**	***P*-value**	**OR; 95% CI**	***P*-value**
Age (years)	1.072; 1.064–1.080	<0.001	0.994; 0.985–1.003	0.196
**Gender**		0.002		0.959
Female	1			
Male	1.276; 1.096–1.487		0.995; 0.824–1.202	
**Ethnicity**		0.458		0.109
Asian	1			
Black	0.789; 0.485–1.283	0.339	0.577; 0.313–1.063	0.078
Hispanic	0.810; 0.450–1.457	0.481	0.945; 0.446–2.003	0.882
White	0.991; 0.649–1.513	0.967	0.868; 0.510–1.478	0.602
**Marital status**		0.479		0.365
Divorced	1		1	
Married	0.835; 0.648–1.076;	0.163	0.897; 0.654–1.230	0.501
Single	0.900; 0.686–1.180	0.445	1.094; 0.777–1.541	0.608
Other	1.027; 0.541–1.952	0.934	0.891; 0.403–1.970	0.775
**ICU type**		0.310		0.157
SICU	1			
TSICU	0.875; 0.677–1.130	0.306	0.951; 0.689–1.311	0.758
MICU	0.871; 0.726–1.044	0.135	0.814; 0.649–1.019	0.073
**Complications**, ***n*** **(%)**
CHF	2.378; 2.022–2.797	<0.001	1.549; 1.274–1.884	<0.001
Peripheral vascular disease	1.051; 0.795–1.389	0.726	1.167; 0.823–1.653	0.386
Chronic pulmonary disease	1.350; 1.139–1.600	0.001	1.531; 1.237–1.897	<0.001
Hypertension	0.955; 0.816–1.117	0.563	1.183; 0.977–1.433	0.086
Diabetes	1.111; 0.944–1.308	0.204	1.194; 0.977–1.460	0.082
Renal failure	1.459;1.203–1.769	<0.001	1.599; 1.254–2.040	<0.001
Chronic liver disease	0.664; 0.531–0.829	<0.001	0.707; 0.539–0.926	0.012
Stroke	3.756; 2.269–6.215	<0.001	5.047; 2.300–11.073	<0.001
SOFA	1.051; 0.795–1.389	0.267	0.979; 0.949–1.011	0.194
**Coagulation disorder**		<0.001		<0.001
Coagulation disorder score = 0	1		1	
Coagulation disorder score = 1 or 2	1.566; 1.295–1.894	<0.001	1.592; 1.261–2.010	<0.001
Coagulation disorder score = 3 or 4	2.022; 1.628–2.510	<0.001	1.780; 1.372–2.309	<0.001
Coagulation disorder score = 5 or 6	2.126; 1.559–2.898	<0.001	1.763; 1.218–2.551	0.003

*AF, atrial fibrillation; CHF, congestive heart failure; SOFA, sequential organ failure assessment; PSM, propensity score matching, with the covariates of age, gender, ICU type, ethnicity and marital status. Multivariate logistic regression analysis includes the factors of age, gender, ethnicity, marital status, ICU type, CHF, peripheral vascular disease, chronic pulmonary disease, hypertension, diabetes, renal failure, chronic liver disease, stroke, SOFA score, and coagulation disorder type*.

### The Interaction of AF and Early Coagulation Disorder on Mortality

With the population before PSM, univariate regression analyses showed that AF was relevant to short-term outcomes, including in-hospital mortality (OR = 1.534; 95% CI: 1.311–1.795; *P* < 0.001), ICU mortality (OR = 1.326; 95% CI: 1.101–1.597; *P* = 0.003), 28-day mortality (OR = 1.510; 95% CI: 1.298–1.757; *P* < 0.001) and 90-day mortality (OR = 1.139; 95% CI: 1.006–1.289; *P* = 0.039). On multivariate logistic regression analyses, AF was an independent risk factor for in-hospital mortality (adjusted OR = 1.323; 95% CI: 1.078–1.624; *P* = 0.008) and 90-day mortality (adjusted OR = 1.243; 95% CI: 1.073–1.441; *P* = 0.004). In septic patients with different coagulation disorder scores, AF was significantly associated with an increased risk of 90-day mortality when coagulation disorder scores were 1 or 2 and 3 or 4 (adjusted OR = 1.291; 95% CI: 1.017–1.639; *p* = 0.036; adjusted OR = 1.355; 95% CI: 1.004–1.828; *p* = 0.047). However, there were no differences in patients with all coagulation scores for in-hospital mortality. Among patients with coagulation scores of 0 and 5 or 6, AF was not associated with 90-day mortality. (Tables 4, 5).

**Table 4:** Univariate and multivariate logistic regression analysis of AF and mortality.

	**Univariate**		**Multivariate**	
	**OR; 95% CI**	***P*-value**	**OR; 95% CI**	***P*-value**
In-hospital mortality	1.534; 1.311–1,795	<0.001	1.323; 1.078–1.624	0.008
ICU mortality	1.326; 1.101–1.597	0.003	1.195; 0.937–1.525	0.150
28-day mortality	1.510; 1.298–1.757	<0.001	1.195; 0.983–1.453	0.074
90-day mortality	1.139; 1.006–1.289	0.039	1.243; 1.073–1.441	0.004

*Multivariate logistic regression analysis includes the factors of age, gender, ethnicity, marital status, ICU type, CHF, peripheral vascular disease, chronic pulmonary disease, hypertension, diabetes, renal failure, chronic liver disease, stroke, SOFA score, and coagulation disorder type*.

**Table 5:** The interaction between AF and early coagulation disorder on morality.

	**In-hospital mortality**		**90-day mortality**	
	**OR; 95% CI**	***p*-value**	**OR; 95% CI**	***p*-value**
Coagulation disorder score = 0	1.417; 0.875–2.296	0.157	1.139; 0.850–1.526	0.383
Coagulation disorder score = 1 or 2	1.340; 0.960–1.869	0.085	1.291; 1.017–1.639	0.036
Coagulation disorder score = 3 or 4	1.242; 0.835–1.849	0.285	1.355; 1.004–1.828	0.047
Coagulation disorder score = 5 or 6	1.187; 0.690–2.045	0.535	1.356; 0.812–2.264	0.245

*Multivariate logistic regression analysis includes the factors of age, gender, ethnicity, marital status, ICU type, CHF, peripheral vascular disease, chronic pulmonary disease, hypertension, diabetes, renal failure, chronic liver disease, stroke, and SOFA score*.

## Discussion

This study suggested an interaction between AF and early coagulation disorder on in-hospital mortality as well as 90-day mortality. In the subgroup analysis stratified by early coagulation disorder scores, AF was associated with an increased risk of 90-day mortality when the scores of early coagulation disorder were 1 or 2 and 3 or 4. The risk of AF was also related to stroke, age, gender, CHF, chronic pulmonary disease, renal failure, and chronic liver disease.

In this study, AF was significantly correlated with higher in-hospital mortality and 90-day mortality than non-AF in sepsis, which is consistent with previous studies (15, 16). Desai et al. (16) reported that although in-hospital mortality and hospital stay decreased between 2010 and 2014 in the sepsis-AF cohort, septic patients with AF had increased all-cause mortality during hospitalization, a longer hospital stay, and higher hospitalization expenses than non-AF individuals. The review also assessed the morbidity of AF and the relationship between AF and adverse events in sepsis. The morbidity of new-onset AF in sepsis was 20.6%. New-onset AF was correlated with excess mortality during hospitalization and 28 days, 1 year as well as 5 years after discharge (15). Therefore, AF is regarded as a marker of disease severity and risk factor of death. The pathogenesis of AF in sepsis can be divided into two steps: the changes of the atrial matrix and trigger events (17, 18). Before the onset of sepsis, patient characteristics, such as advanced age, diabetes, acute renal impairment, and CHF, lead to atrial structure and electrical remodeling through the production of TGF-β, angiotensin, ROS, inflammatory factors, and changes in intracellular ion channels. During sepsis, infection, overloading volume, and thyroid crisis can further trigger AF. In addition, AF is connected with decreased cardiac output and increased cardiac afterload. AF can also aggravate atrial cardiomyopathy, further increasing the risk of thromboembolism. Thus, AF and its adverse consequences dramatically increase short-term and long-term mortality.

AF is correlated with an increased risk of thrombosis (19), but it is unknown whether early abnormal coagulation function is a risk factor for AF. The present studies indicate that sepsis is associated with early coagulation disorder, including a fall in platelet count and lengthening of clotting time. SIC is used to identify the earlier stage of DIC in sepsis, which was proposed by the Scientific and Standardization Committee on DIC (20). The main mechanisms contributing to SIC and DIC consist of coagulation initiation, activation of platelets, increased inflammatory cells, and damage to vascular endothelium (8, 10, 21) due to damage-associated molecular patterns (DAMPs) (22, 23), neutrophil extracellular traps (NETs) (24), extracellular vehicles (EVs) (25) and endothelial glycocalyx damage (26). It is worth noting that vascular endothelial dysfunction and anticoagulant/fibrinolytic disorder are major markers of SIC. Vascular endothelium connects with membrane-binding proteoglycans and glycosaminoglycan side chains which can bind to antithrombin so that thrombosis is negatively affected in normal circumstances. However, the inhibition weakens or disappears when the vascular endothelium is damaged. Endothelial cells also have a vital influence on fibrinolysis through tissue-type plasminogen activators and their inhibitors, while the balance is disrupted and tends to inhibit fibrinolysis during sepsis (13). Thus, derangement of the coagulation/fibrinolytic system is a hallmark of SIC. It is speculated that abnormal coagulation function caused by sepsis is associated with a disturbance in hemodynamics (23), which can contribute to the development of AF.

In sepsis, there are a large proportion of patients exhibiting DIC, which is diagnosed by coagulation indicators of prothrombin time, platelet count, fibrinogen, D-dimer and fibrin degradation products. However, the DIC scoring system is only suitable for the evaluation of severe coagulation dysfunction (8). To provide an early and valuable prediction of coagulation disorder, this work defines early coagulation disorder with reference to SIC and coagulopathy. This is because SIC provides an earlier diagnose and includes most cases of DIC. Septic patients with SIC can also benefit from anticoagulant therapy even though they do not meet the criteria for DIC (23). In addition, sepsis-associated DIC is characterized by excessive inhibition of fibrinolysis due to overproduction of plasminogen activator inhibitors. Therefore, hypofibrinogenemia is uncommon and fibrin-related markers are not correlated with the severity of illness. In contrast, platelet count and PT play a vital role in the mortality of septic patients. Therefore, PLT, INR, and APTT were selected as common and major coagulation indicators to evaluate early coagulation dysfunction (27).

Currently, there are few models to predict the risk of AF. Based on the findings of our work, early coagulation status should be considered, in part, as a clue of AF risk in septic patients and may be regarded as an indicator of the AF prediction model accompanied with other indicators (27). Because of the poor prognosis in septic patients with AF, this model is vital for the risk stratification to promote the early management of these patients. However, a retrospective study in these patients makes robust conclusions difficult. Therefore, more prospective, multicenter trials to evaluate the relationship between early coagulation and AF are necessary.

### Limitations

Moreover, we acknowledge certain limitations in this work. First, it was a retrospective study covering hospital admissions of more than 10 years. During this period, the decision-making process of AF and sepsis management developed dramatically, which is a confounding factor affecting mortality (17, 28). Second, information on medication in septic patients before the onset of AF, such as glucocorticoids and vasopressin, was not available (29). Owing to the influence of these medications on AF morbidity, the relationship between coagulation disorders and AF can interfere. Third, we have only taken coagulation analysis with PLT, INR, and APTT. Given the retrospective nature of our study, clinical information such as other coagulation factors and fibrinogen were not available, so further analysis about the association between coagulation and AF cannot be fully explored. Finally, given the retrospective nature of the study, clinical information on several aspects of AF and sepsis in the cohort, such as AF loading assessment, AF classification and treatment, were not be fully available.

### Future Directions

The present results highlight the influence of early coagulation dysfunction on septic patients with AF. However, larger, prospective trials are necessary to determine the relationship between coagulation disorder and AF. This study may provide the basis for future research on the relationship between early coagulation and AF.

## Conclusion

Coagulation disorder within the first 24 h after admission to the ICUs is related to a higher risk of AF in individuals with sepsis. AF is an independent risk factor of in-hospital mortality and 90-day mortality in sepsis, and the effect of AF on 90-day mortality varies with the severity of early coagulation disorder. It is of great importance for clinicians to make personalized management for septic patients with AF based on their early coagulation status.

## Data Availability Statement

The original contributions presented in the study are included in the article/Supplementary Material, further inquiries can be directed to the corresponding author/s.

## Ethics Statement

Ethical review and approval was not required for the study on human participants in accordance with the local legislation and institutional requirements. Written informed consent for participation was not required for this study in accordance with the national legislation and the institutional requirements.

## Author Contributions

YL, YT, RF, and JD: methodology, writing, and revision. YL, RM, XC, JW, and JS: data curation and investigation. GL and CL: supervision, reviewing, and editing the manuscript. All authors contributed to the article and approved the submitted version.

## Conflict of Interest

The authors declare that the research was conducted in the absence of any commercial or financial relationships that could be construed as a potential conflict of interest.

## Publisher's Note

All claims expressed in this article are solely those of the authors and do not necessarily represent those of their affiliated organizations, or those of the publisher, the editors and the reviewers. Any product that may be evaluated in this article, or claim that may be made by its manufacturer, is not guaranteed or endorsed by the publisher.
