# Supplementary material for: Early Coagulation Disorder Is Associated With an Increased Risk of Atrial Fibrillation in Septic Patients
Source: Front Cardiovasc Med. 2021 Sep 30;8:724942. doi: 10.3389/fcvm.2021.724942 (PMC8514978; doi:10.3389/fcvm.2021.724942)
Supplement: Supplementary file 1 [file Table_1.docx]

| Supplementary Table 1 Demographic characteristics between AF group and Non-AF group within the first 24 hours of ICUs after PSM. | | | | |
| --- | --- | --- | --- | --- |
| Variables | Total | Non-AF | AF | *P*-value |
|  | n=2078 | n=1039 | n=1039 |  |
| Age (years) | 69 (62-76) | 69 (61-75) | 69 (62-75) | 0.750 |
| Female, n (%) | 767 (36.91) | 381 (36.67) | 386 (37.15) | 0.820 |
| Ethnicity, n (%) |  |  |  | 0.568 |
| Asian | 61 (2.94) | 29 (2.79) | 32 (3.08) |  |
| Black | 194 (9.34) | 100 (9.62) | 94 (9.05) |  |
| Hispanic | 57 (2.74) | 26 (2.50) | 31 (2.98) |  |
| White | 1562 (75.17) | 772 (74.30) | 790 (76.03) |  |
| Other | 204 (9.82) | 112 (10.78) | 92 (8.85) |  |
| BMI (kg/m^2^) | 27.7 (24.0-32.9) | 26.9 (23.4-31.5) | 28.7 (24.7-34.2) | <0.001 |
| Insurance type, n (%) |  |  |  | 0.036 |
| Government | 26 (1.25) | 14 (1.35) | 12 (1.15) |  |
| Medicaid | 122 (5.87) | 75 (7.22) | 47 (4.52) |  |
| Medicare | 1378 (66.31) | 662 (63.72) | 716 (68.91) |  |
| Private | 540 (25.98) | 283 (27.24) | 257 (24.74) |  |
| Self-pay | 12 (0.58) | 5 (0.48) | 7 (0.67) |  |
| Marital status, n (%) |  |  |  | 0.199 |
| Single | 515(24.78) | 239(23.00) | 276(26.56) |  |
| Married | 1334(64.20) | 690(66.41) | 644(61.98) |  |
| Divorced | 199(9.58) | 95(9.14) | 104(10.01) |  |
| Other | 30(1.44) | 15(1.44) | 15(1.44) |  |
| ICU type, n (%) |  |  |  | <0.001 |
| SICU | 455 (21.90) | 218 (20.98) | 237 (22.81) |  |
| TSICU | 252 (12.13) | 128 (12.32) | 124 (11.93) |  |
| MICU | 1371 (65.98) | 693 (66.70) | 678 (65.26) |  |
| Vital signs |  |  |  |  |
| *Supplementary Table 1 continued* | | | | |
| HR (bpm) | 88 (76-100) | 87 (76-97) | 89 (78-102) | 0.017 |
| RR (bpm) | 20 (17-23) | 19 (17-22) | 20 (17-23) | 0.061 |
| T (°C) | 36.9 (36.4-37.4) | 36.9 (36.5-37.4) | 36.8 (36.4-37.4) | 0.042 |
| MAP (mmHg) | 75 (69-83) | 75 (69-84) | 75 (69-82) | 0.226 |
| Complications, n (%) |  |  |  |  |
| Hypertension | 1088 (52.36) | 505 (48.60) | 583 (56.11) | 0.001 |
| Diabetes | 662 (31.86) | 292 (28.10) | 370 (35.61) | <0.001 |
| CHF | 715 (34.42) | 292 (28.13) | 423(40.71) | <0.001 |
| Peripheral vascular disease | 158 (7.61) | 65 (6.26) | 93 (8.95) | <0.001 |
| Renal failure | 427 (20.56) | 164 (15.80) | 263 (25.31) | <0.001 |
| Chronic liver disease | 333 (16.03) | 190 (18.30) | 143 (13.76) | 0.005 |
| Chronic pulmonary disease | 516 (24.84) | 213 (20.52) | 303 (29.16) | <0.001 |
| Stroke | 45 (2.17) | 8 (0.77) | 37 (3.56) | <0.001 |
| DIC | 44 (2.1) | 24 (2.3) | 20 (1.9) | 0.542 |
| Critical illness score |  |  |  |  |
| ECI | 14（7-20） | 11（5-17） | 16（11-22） | <0.001 |
| SOFA | 5 (3-7) | 5 (4-8) | 5 (3-8) | 0.643 |
| APSIII | 51 (40-65) | 50 (40-65) | 53 (42-67) | 0.009 |
| SAPSII | 41 (33-50) | 41 (33-50) | 41 (34-51) | 0.456 |
| OASIS | 34 (29-41) | 34 (28-40) | 35 (29-42) | 0.009 |
| GCS | 15 (13-15) | 15 (13-15) | 15 (13-15) | 0.542 |
| Coagulation function |  |  |  |  |
| PLT（10^9/L） | 181 (110-257) | 173 (88-254) | 176 (106-255) | 0.726 |
| INR | 1.4 (1.2-1.9) | 1.4 (1.2-1.7) | 1.5 (1.2-2.3) | <0.001 |
| APTT (s) | 33.0 (27.0-43.2) | 34.1 (28.5-48.6) | 34.6 (29-46.0) | 0.017 |
| Outcomes |  |  |  |  |
| ICU morality, n (%) | 260 (12.51) | 132 (12.70) | 128 (12.32) | 0.791 |
| In-hospital mortality, n (%) | 379 (18.24) | 183 (17.61) | 196 (18.86) | 0.460 |
| *Supplementary Table 1 continued* | | | | |
| 28-day mortality, n (%) | 421 (20.26) | 210 (20.21) | 211 (20.31) | 0.956 |
| 90-day mortality, n (%) | 966 (46.49) | 347 (33.40) | 619 (59.58) | <0.001 |
| AF, atrial fibrillation; BMI, body mass index; SICU, surgical intensive care unit; TSICU, trauma surgical intensive care unit; MICU, medical intensive care unit; HR, heart rate; RR, respiratory rate; T, temperature; MAP, mean arterial pressure; CHF, congestive heart failure; DIC, disseminated intravascular coagulation; ECI, Elixhauser comorbidity index; SOFA, sequential organ failure assessment; APSIII, acute physiological score III; SAPSII, simplified acute physiology score II ; OASIS, overall anxiety severity and impairment scale; GCS, Glasgow coma score; PLT, platelet count; INR, international normalized ratio; APTT, activated partial thromboplastin time. | | | | |
